# Multidrug-resistant* Staphylococcus aureus* Colonization in Healthy Adults Is more Common in Bhutanese Refugees in Nepal than Those Resettled in Ohio

**DOI:** 10.1155/2019/5739247

**Published:** 2019-07-01

**Authors:** Jhalka Kadariya, Dipendra Thapaliya, Sabana Bhatta, Ram Lal Mahatara, Sandra Bempah, Nabin Dhakal, Tara C. Smith

**Affiliations:** ^1^Kent State University, College of Public Health, Department of Biostatistics, Environmental Health Sciences and Epidemiology, 750 Hilltop Drive, Kent, OH 44242, USA; ^2^AMDA Hospital, Madhumalla Road, Damak, Jhapa, Nepal; ^3^International Organization for Migration (IOM), Bhrikuti Chwok, Damak, Jhapa, Nepal

## Abstract

Although studies have shown that human migration is one of the risk factors for the spread of drug-resistant organisms such as methicillin-resistant* Staphylococcus aureus* (MRSA), surveillance studies examining MRSA among refugee populations in the US are lacking. This study aimed to assess the prevalence and molecular characteristics of* S. aureus *among Bhutanese refugees living in Nepal and resettled in Northeast Ohio (NEO). One hundred adult Bhutanese refugees from each geographic location were enrolled between August 2015 and January 2016. The participants were interviewed to collect demographic information and potential risk factors for carriage. Nasal and throat swabs were collected for bacterial isolation. All* S. aureus* isolates were characterized by* spa* typing and tested for the presence of Panton-Valentine leukocidin (PVL) and* mec*A genes; selected isolates were tested by multilocus sequence typing (MLST). The overall prevalence of* S. aureus* was 66.0% and 44.0% in NEO and Nepal, respectively. In Nepal, 5.8% (3/52) of isolates were MRSA and 1.1% (1/88) in NEO. Twenty-one isolates in NEO (23.9%) were multidrug-resistant* S. aureus* (MDRSA), while 23 (44.2%) in Nepal were MDRSA. In NEO, 41* spa *types were detected from 88* S. aureus* isolates. In Nepal, 32* spa* types were detected from 52* S. aureus* isolates.* spa* types t1818 and t345 were most common in NEO and Nepal, respectively. The overall prevalence of PVL-positive isolates among* S. aureus* in Nepal and NEO was 25.0% and 10.2%. ST5 was the most common sequence type in both locations. Bhutanese refugees living in Nepal and resettled in NEO had high prevalence of* S. aureus* and MDRSA. The findings suggest a potential need for CA-MRSA surveillance among the immigrant population in the U S and among people living in Nepal, and a potential need to devise appropriate public health measures to mitigate the risk imposed by community-associated strains of* S. aureus *and MRSA.

## 1. Introduction

*Staphylococcus aureus *is a common opportunistic bacterium. This bacterium is an important pathogen due to combination of toxin-mediated virulence, invasiveness, and antibiotic resistance [1]. Although it may be part of the normal human microbiota, it can cause wide range of diseases from skin and soft-tissue infections (STIs) to severe invasive disease such as infective endocarditis, osteomyelitis, and toxic shock syndrome [2].* S. aureus *is also a major cause of food-borne illness worldwide [3]. An estimated 30% and 1.5% of the US population is colonized with methicillin-susceptible* Staphylococcus aureus *(MSSA) and methicillin-resistant* Staphylococcus aureus *(MRSA), respectively [4, 5].* S. aureus *can be recovered from many locations in and on the body, including the nose, throat, axillae, and groin, but the most important site for colonization is thought to be the anterior nares (nostrils) [4]. While colonization with* S. aureus *itself does not harm the host, colonization is a risk factor for developing subsequent symptomatic infections [6].

*S. aureus *accounts for nearly 20% of bloodstream infections in the hospital setting [7]. The treatment of* S. aureus *infections is often challenging due to the emergence of multidrug-resistant strains [8, 9]. Although drug resistance has been seen as a major risk in healthcare settings, a similar increasing trend is observed in community-acquired infections [10].

Traditionally regarded as a nosocomial pathogen [11], MRSA infection outside of hospital has increased in incidence over the last decade [12] and has emerged as a major public health concern worldwide [13]. For example, community-acquired methicillin-resistant* S. aureus *(CA-MRSA) is the leading cause of identifiable skin and soft-tissue infections (SSTIs) observed in US emergency rooms [14]. An estimated 80,461 invasive MRSA infections occurred nationally in 2011. Of these, 16,560 were community-associated infections [15]. The economic impact of CA-MRSA is tremendous. A recent publication suggested these infections impose an annual burden of $478 million -$2.2 billion on third-party payers and $1.4 billion -$13.8 billion on society in the US [16]. Because of lengthy hospital stays, increased costs, and higher mortality, MRSA infections have imposed an increased disease burden in the last decade [12, 17].

The disease burden caused by* S. aureus *is poorly understood in developing countries [18]. Although there have been some studies conducted in the context of Nepal, very little is known about the molecular epidemiology and transmission dynamics of* S. aureus *in this country. Most of the studies conducted in Nepal have been focused on the hospital environment, examining only the prevalence and phenotypic characterization of* S. aureus* [19–24]. As such, the molecular epidemiology of* S. aureus* in community settings has not been investigated, and* S. aureus *colonization parameters among the general population or specific groups such as Bhutanese refugees is unknown.

In the US, little is known regarding the prevalence and epidemiology of MRSA in immigrant populations. Although some infections, including tuberculosis and syphilis, may be detected during the pre- or postarrival screening, MRSA colonization is not examined, as there has been no policy or protocol for such screening by the US health institutions.

In the past several years, the US has granted asylum to Bhutanese immigrants of Nepalese origin that were displaced from Bhutan due to political reasons. As a result, Bhutanese of Nepali origin were one of the largest groups of refugees resettled in the US, accounting for 19% of the total 322,565 refugees admitted into the US between 2008 and 2012 [25]. This study investigated the prevalence and molecular epidemiology of S*. aureus *carriage in adult Bhutanese refugees living in camps in eastern Nepal and resettled in Northeast Ohio (NEO), United States.

## 2. Materials and Methods

### 2.1. Study Population

A community-based cross-sectional study was conducted among Bhutanese refugees living in Nepal and NEO in between August 2015 and January 2016. The study sites were Beldangi refugee camp in Jhapa district of Nepal and Akron and Cleveland metropolitan area in NEO. A convenience sample of 100 Bhutanese refugees aged 18 years and older, upon their consent to participate in the study, was recruited in each site. In both study sites, participants were recruited in community settings. Most of the participants in Nepal were enrolled in their home. A small portion of participants were invited to come to Primary Health Center for interview and sampling for their convenience. However, a meeting room was used for sampling. In NEO, the participants were recruited by visiting Nepalese grocery stores, Nepali restaurants, and the households of the participants and via distribution of flyers in the community.

### 2.2. Survey Instrument and Study Variables

Data obtained from the questionnaire included sociodemographic information such as family size, education, income, and occupation; medical information such as presence or absence of asthma, heart disease, diabetes, autoimmune disease, and cancer; presence or absence of risk factors such as recent history of hospitalization, hospital visit, history of antibiotic treatment, time spent in jail, participation in team or contact sports, ownership of pets, and history of* S*.* aureus *or MRSA infection in the respondent or his/her family members; occupational exposure; and some miscellaneous exposures such as exposure to manure, pork, chicken, turkey meats, live animals, and types of soap used in home. An outcome variable “*S. aureus *and MRSA colonization positive” was assessed in association with exposure variables such as environmental and occupational exposures.

### 2.3. Data Collection

A set of pretested semistructured questionnaires was used to collect information from study participants. A face-to-face interview was conducted with each participant by the research team. Questionnaires, first developed in English language were translated into Nepali language, and the Nepali language questionnaire set was used to interview the participants in both study sites. The purpose of the study, its potential harm, and benefits were explained to each participant. Verbal and written consent was obtained from each participant to participate in the study. A copy of Nepali consent form was provided to each participant to take with them. The Institutional Review Board (IRB) of Kent State University and of the Nepal Health Research Council (NHRC) approved the study.

### 2.4. Sample Collection and Bacterial Culture

Biological specimens were collected from nares and throats using sterile swabs as described previously [26] and transported to the laboratory on blue ice packs. Samples were processed within 24 hours of collection. Samples were inoculated into 5ml (1X concentration) Baird Parker Broth with tellurite enrichment (Sigma products-Sigma-Aldrich, St. Louis, MO) in a 15 ml sterile plastic tubes by twisting the swab vigorously and incubated for 24 hours at 37°C. One microliter cultured broth was inoculated onto Baird Parker agar (BPA) with EY tellurite enrichment (BD) and was incubated for 48 hours at 37°C. Isolates were subcultured onto Columbia colistin-nalidixic agar with 5% sheep blood (Columbia CNA; Ramel) and tested via the catalase test, coagulase test, and using a* S. aureus* latex agglutination assay (Pastorex Staph-plus, Bio-Rad). All* S. aureus *isolates were stored at -80°C.* S. aureus* prevalence was calculated as a percent of all individuals tested who were positive at either carriage site (nose or throat); MRSA and MDRSA prevalence were calculated as percentage of all* S. aureus* isolates who demonstrated these phenotypes.

### 2.5. Antimicrobial Susceptibility Testing (AST)

*S. aureus *isolates were tested for antibiotic susceptibility via Vitek-2 System version R06.01 (BioMérieux, Durham, NC) per manufacturer's instructions. Isolates were tested for susceptibility to benzylpenicillin, oxacillin, tetracycline, erythromycin, ciprofloxacin, moxifloxacin, minocycline, clindamycin, trimethoprim-sulfamethoxazole (TMS), quinupristin/dalfopristin, gentamicin, levofloxacin, linezolid, daptomycin, vancomycin, rifampin, minocycline, tigecycline, and nitrofurantoin per Clinical and Laboratory Standards Institute guidelines [27]. Bacterial isolates resistant to three or more antibacterial or resistant to oxacillin were designated as multidrug-resistant* S. aureus* (MDRSA) [28].

### 2.6. Molecular Characterization

Genomic DNA was extracted using the Wizard Genomic DNA preparation kit (Promega, Madison, WI). The staphylococcus protein A (*spa*) gene was amplified as described [29, 30]. The detection of the* mecA *and PVL genes was determined by PCR [31, 32].* spa *types were assigned using Ridom® StaphType software (version2.2.1; Ridom GmbH, $W\ddot{\overset{-}{u}}rzburg$, Germany). The Based upon Repeat Pattern (BURP) algorithm was used to group* spa* types into genetic clusters based on their genetic proximity [33]. A selected number of isolates were subjected to multilocus sequence typing (MLST) as described previously [34]. The most common, unique, and/or isolates of interest were chosen for MLST (e.g., those which were present in high numbers, low numbers, or unique in some manner including a novel* spa* type). Sequence types (STs) were assigned using organism specific MLST database (https://pubmlst.org/saureus/). PHYLOViZ software v2.0 was used to analyze Global optimal eBURST of STs and to draw minimum spanning tree and relatedness of STs as described by Francisco et al. [35, 36]. Positive (USA 300) and negative controls (reaction mixture without DNA template) were used in* mec*A, PVL, and* spa* PCR.

### 2.7. Statistical Analysis

Frequency distribution and proportions were calculated for the categorical variables while mean (± standard deviation), median, and range were calculated for continuous variables. Chi- square test or Fisher's exact test and logistic regression were used to assess the association between outcome variables and the potential risk factors. The crude odds ratio (OR) was calculated with 95% confidence intervals. Statistical significance was assessed at *α* = 0.05 level. All statistical analyses were conducted using SAS statistical software (Version 9.3, SAS Institute Inc., Cary, NC).

## 3. Results

### 3.1. Demographic Information

A total of 400 samples (200 nasal and 200 throat) were collected from 200 participants (100 from each study site). The mean age of the participants was 30.7 (SD 10.9) years in NEO and 36.8 (SD 13.3) years in Nepal. In NEO, 58% and 42 % of the participants were male and female, respectively, while the percentage of male and female participants in Nepal was 29% and 71%, respectively. In Nepal, the majority of the participants was from the Kirat religion (79%), married (86%), and had a formal education (60%). In NEO, the majority of the participants had formal education (86%) (Table 1).

### 3.2. Prevalence of* S. aureus*

The overall prevalence of* S. aureus* was 66.0% (66/100; 95% CI 56.7%–75.3%) and 44.0% (44/100; 95% CI 34.3%–53.7%) in NEO and Nepal, respectively, for individuals colonized at either biological site. A statistically significant difference (p=0.002) was observed in the prevalence of* S. aureus* between the two sampling sites.

In Nepal, 39.0% (39/100; 95% CI 29.4%–48.5%) and 13.0% (13/100; 95% CI 6.4%–19.6%) of the participants were colonized with* S. aureus* in their throat and nose, respectively, while the prevalence of* S. aureus* colonization in nose and throat in NEO was 25.0% (25/100; 95% CI 16.5%–33.5%) and 63.0% (63/100; 95% CI 53.5%–72.5%), respectively. The difference of* S. aureus* colonization in nose and throat between Nepal and NEO was significant (p = 0.04 for nose and 0.001 for throat). The prevalence of positive isolates from both locations (nose and throat) from the same participants was 16% (7/44; 95% CI 5.1% - 26.7%) in Nepal and 33% (22/66; 95% CI 22% - 44.7%) in NEO. This difference was significant (= 0.04).

The overall prevalence of MRSA (based on the presence of the* mec*A gene) was 3.0% (3/100; 95% CI 0 – 6.3%) in Nepal and 1.0% (1/100; 95% CI 0 – 2.9%) in NEO. No significant difference was observed in MRSA prevalence between Nepal and NEO (p = 0.62, OR 0.32; 95% CI 0.03%–3.19%).

Tables 2 and 3 show participants' demographics as well as medical, occupational, and environmental risk factors previously associated with the presence or absence of* S. aureus*. In NEO, a higher prevalence of* S. aureus* was found among males, participants who handled meat (pork and poultry) in the last six months, and participants who had skin infections in the last six months. In Nepal, a higher prevalence of* S. aureus* was found among females, participants who had children attending day care, and found who were diagnosed with skin infections and autoimmune diseases (Tables 2 and 3).

### 3.3. Antibiotic Susceptibility Testing

**Figure 1:**
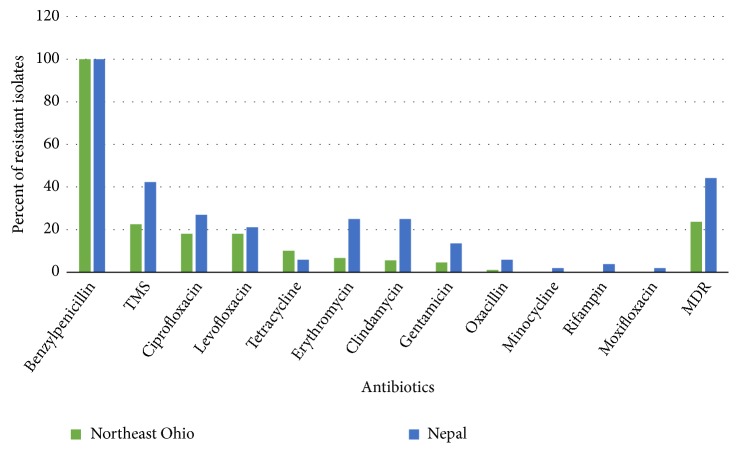
Antibiotic resistance profile of isolates. MDR, multidrug-resistant.

All isolates were tested for antibiotic susceptibility. All isolates in Nepal and NEO were resistant to penicillin. Oxacillin resistance was observed for 1.1% (1/89) and 5.8% (3/52) of the isolates in NEO and Nepal, respectively. Resistance to other antibiotics was higher for Nepal than Ohio. Additionally, MDRSA rates were higher in Nepal than Ohio (44.2% and 23.6%, respectively) (Figure 1).

### 3.4. Molecular Characterization of* S. aureus*

*spa* typing was carried out on all positive isolates. In NEO, a total of 41* spa *types were detected from 88* S. aureus* isolates. Overall, t1818 was the most common* spa* type (12/88; 13.6%), followed by t002 (7/88; 8.0%), t104 (6/88; 6.8%), t008, t084, t085, t091 (4/88 each; 4.5%), and t521 (3/88; 3.4%). All other* spa* types (t005, t10559, t1094, t11906, t122, t127, t131, t16153, t164, t1654, t1818, t1839, t190, t1911, t1931, t2119, t213, t2379, t2663, t273, t345, t4371, t442, t491, t521, t6127, t616, t688, t7264, t774, t803, t818, t878, t934, and t9432) were ≤ 2.2% of* S. aureus* isolates.

In Nepal, a total of 32* spa *types were detected from 52* S. aureus* isolates. Overall, t345 was the most common* spa* type (5/52; 9.6%), followed by t311, t2119 (4/52 each; 7.7%), t442, t127 (3/52 each; 5.8%), t021, t084, t091, t159, t164, and t934 (2/52 each; 3.8%). All other* spa* types (t002, t008, t085, t1149, t11917, t12219, t1427, t15347, t15578, t15579, t15580, t15581, t1839, t2663, t304, t3175, t376, t4188, t701, t7766, t878) were less than 2.0% of the isolates.

*spa* types were grouped based on their genetic proximity to* spa* types associated with specific cluster complexes. BURP grouping using default parameters (“exclude parameters that are shorter than 5 repeats” and “*spa* types are clustered if costs are less or equal than 4”) resulted in 7* spa* CCs and 11 singletons (t021, t127, t159, t164, t878, t1149, t2663, t11917, t15579, t15580, and t15581) in Nepal and 6* spa* CCs and 16 singletons (t005, t091, t104, t122, t127, t164, t190, t213, t616, t818, t878, t1654, t2663, t7264, t10559, and t16153) in NEO (Supplemental Figures 1-3).

Thirty-nine* S. aureus* isolates from NEO and 29 from Nepal were subjected to MLST. A total of 26 different STs were detected from NEO. The most common ST was ST5 (17.9%; 7/39), followed by ST8 (10.3%; 4/39), ST1, ST15, ST3206, and ST96 (5.1% each; 2/39). No other STs constituted more than one tested* S. aureus* isolates (Table 4). A total of 22 different STs were detected from Nepal. The most common ST was ST5 (10.3%; 3/29), followed by ST15, ST3206, ST6, ST672, and ST80 (6.9 % each; 2/29). No other STs constituted more than one tested* S. aureus* isolate (Table 5).

The overall prevalence of PVL genes among* S. aureus* isolates in Nepal and NEO was 25.0% (13/52; 95% CI 13.2%–36.7%) and 10.2% (9/88; 95% CI 3.8%-16.4%), respectively. A statistically significant higher prevalence of PVL-positive isolates was observed in Nepal compared to NEO (p=0.02). All PVL-positive* S. aureus* (t1839, t345, t1094, t2663, t008, t213, t11906, and t005) from NEO were MSSA. Two-thirds of the PVL-positive isolates from NEO (66.7%; 6/9) and 46.2% (6/13) from Nepal were MDRSA. Only one PVL-positive isolate from NEO (t345) was* mec*A-positive. Three* mec*A-positive isolates from NEO belonged to ST1 (t127), ST6 (t304), and ST3206 (t345). One* mec*A-positive isolate from Nepal belonged to ST8 (t008). All* mec*A-positive isolates were also phenotypically resistant to oxacillin.

## 4. Discussion

In this cross-sectional study, we investigated the prevalence and molecular epidemiology of* S. aureus* among Bhutanese refugees living in Nepal (n=100) and in NEO (n=100). The overall prevalence of* S. aureus* was lower in Nepal (44.0%) compared to NEO (66.0%). The* S. aureus* and MRSA prevalence of this study in Nepal is similar to previous studies that documented between 15.7%–43.8%* S. aureus* carriage rates and 2.3%–7.5% MRSA nasal colonization among healthcare workers and patients in Nepal [37–42]. However, the nasal carriage of* S. aureus* (13.0%) and MRSA (1.0%) in Nepal is lower than in a prior study conducted in a community setting in Nepal [43]. A previous study examining the prevalence of MRSA in a school children in Pokhara City of Nepal via nasal swab documented 17.4% (32/184) overall MRSA prevalence [43]. Such differences in* S. aureus* and MRSA prevalence may be due to geographical, population, and methodological differences. For example, we defined MRSA based on the presence of* mec*A gene, our sampling took place in eastern Nepal, and participants had to be at least 18 years old to be eligible for our study. Work by Rijal et al. (2008) took place in western Nepal, enrolled only minors (younger than 15 years old), and used a phenotypic method (Kirby-Bauer) for MRSA designation. Although the prevalence of MRSA in NEO is consistent with a previous report that suggested 0.84% of the noninstitutionalized US population were colonized with MRSA, the prevalence of* S. aureus* is significantly higher in this study (31.6% versus 66.0%) compared to that report [5].

*S. aureus* prevalence in our study was not associated with demographic factors such as age, gender, marital status, occupation, education, and income in both locations, nor with reported previous antibiotic exposure. The odds of* S. aureus* colonization among participants in NEO was 2.5 times higher than colonization in Nepal (OR = 2.58, 95% CI: 1.45%–4.58%, p <0.001). However, variability in participants' demographic characteristics as well as length of residency in the US may have contributed to these differences. For example, the demographic composition of participants from Nepal was different from those in NEO in regard to gender, religion, marital status, and education. No significant difference was observed in* S. aureus* colonization among participants who lived less than 5 years in NEO.

The prevalence of* S. aureus* in the nose and throat was higher in NEO than in Nepal (p=<0.05). Although the anterior nares are considered as the most common site of* S. aureus* colonization [6], the result of this study showed that the colonization of* S. aureus* was higher in throat compared to the nose in both locations of the study. This result is in consistent with a previous study done in Sweden that found throat as more common site of colonization [44].

Of the 18 drugs tested, resistance was observed to 9 and 12 different antibiotics in NEO and Nepal, respectively (Figure 1). Compared to NEO, a significantly higher proportion of isolates (23.6% versus 44.2%) were MDRSA in Nepal. The higher proportions of isolates were resistant to different classes of antibiotics in Nepal as compared to NEO.* S. aureus* were frequently resistant to drugs that are used commonly in Nepal. For instance, ciprofloxacin is one of the most commonly prescribed antibiotics in Nepal [45], and trimethoprim/sulfamethoxazole is one of the most preferred antimicrobial in Nepal due to its wide spectrum and low cost [46]. One of the studies done in a tertiary care hospital in western Nepal demonstrated that approximately 56% and 27% of the MRSA isolates were resistant to trimethoprim/sulfamethoxazole and ciprofloxacin, respectively [24]. In Nepal, 42% and 27% of the isolates were resistant to trimethoprim/sulfamethoxazole and ciprofloxacin, respectively. Antibiotics overuse is a common drug use problem in Nepal [47]. For example, a study conducted in eastern Nepal documented that 84% of the prescriptions contained antibiotics comprising 42.8% of the total number of drugs [48]. In NEO, 22.5% and 18.0% of the isolates were resistant to trimethoprim/sulfamethoxazole and ciprofloxacin, respectively.

The prevalence of PVL-positive isolates was significantly higher among refugees living in Nepal compared to NEO (p=0.02). This is consistent with prior publications demonstrating a high prevalence PVL-positive isolates in clinical specimens in Nepal [49, 50].

*spa* type t345 (ST 3206) was the most common* spa* type in Nepal.* spa *type t345 was previously isolated from patients infected with* S. aureus* in Banglore, India [51].* spa* type t1818 (ST96) was most common in NEO.* spa* type t1818 has been previously isolated from clinical specimens in France [52], clinical samples from intensive care units of a tertiary hospital in Korea [53], and clinical samples from intensive care unit patients in the Netherlands [54].* Spa* type t345 was found in both locations. However,* spa* type t1818 was found only in NEO. Only one isolate of* spa* types t002 (ST5) and t008 (ST8) each was found in Nepal. In NEO, 7 (8.0%; 7/88) and 4 (4.5%; 4/88) isolates were t002 and t008, respectively.* spa* type t002 (ST5/USA100) and t008 (ST8/USA300) are common hospital-associated and community-associated* S. aureus* stain, respectively. ST5 was the most common sequence type in both locations.

There are number of limitations to this study. This study employed a convenience sampling method to collect a limited number of human samples from populations in narrow geographic locations. As such, the generalizability of these findings is limited. Since the study design is cross-sectional, it does not allow for causal inference between exposures and outcomes. Although most of the medical and occupational risk factors were associated with* S. aureus *colonization, the association was not statistically significant. Hence, the study was not able to establish the demographic as well as medical and occupational exposures potentially implicated with* S. aureus* colonization. Since the study participants in Nepal and NEO are different and resettled refugees had varying duration of time regarding their residency in NEO, there might be other confounding factors affecting* S. aureus* colonization. The lack of longitudinal follow-up limits the understanding of the dynamics of persistent colonization of* S. aureus* and MRSA in the study population as well as the effects of environmental and occupational exposure over time.

The strengths of the study include the acquisition of samples from two locations that enrolled participants from the same ethnocultural heritage. The inclusion of survey questions in addition to human samples provided information concerning exposure variables. To our knowledge, this is the first study conducted to assess the prevalence and molecular characterization of* S. aureus *in immigrant population in the United States and in a community setting in Nepal. Follow-up studies could examine changes in colonization in a longitudinal manner as immigrants are resettled in Ohio or other areas in the US.

## 5. Conclusions

In conclusion, the findings of this study indicate that Bhutanese refugees living in Nepal aand resettled in NEO had high prevalence of* S. aureus* and MDRSA carriage.* S. aureus* colonization in throat was significantly higher than nasal colonization in both locations. The prevalence of MSSA was higher among resettled Bhutanese refugee living in NEO as compared to those living in Nepal. Information about* S. aureus* epidemiology in developing countries is important, as almost 1 billion people cross international boundaries each year. Population mobility imposes threats to the distribution of antimicrobial drug-resistant organisms [10]. MRSA has been isolated from immigrant people in Europe [55, 56]. For example, a total of 56 persons who had recently arrived in Sweden between 2002 and 2003 as immigrants had cases of MRSA, giving an overall risk of 15.9 cases/100,000 immigrants [56]. In Spain, of the 19 patients infected with CA-MRSA, 15 were immigrants from South America [55]. The findings of this study emphasize the potential need for surveillance among immigrant population in the US and among people living in Nepal and a potential need to devise public health interventions to mitigate the risk imposed by* S. aureus *infections.

## Figures and Tables

**Table 1:** Socio-demographic characteristics from NEO and Nepal.

Category		NEO (n=100)	Nepal (n=100)	P-value
Age in years (mean, SD)		30.7, 10.9	36.8, 13.3	
Gender	Female	42	71	<0.05
	Male	58	29	
Religion	Kirat	45	79	<0.001
	Others	55	21	
Marital Status	Married	45	86	<0.001
	Others	55	14	
Education	No formal Ed	14	40	<0.001
	Had formal Ed	86	60	

ED, education.

**Table 2:** Exposure variables in association with *S. aureus *colonizationin NEO (n=100).

Variables	SA+ (%)	SA – (%)	P-value
*Gender*			
Male	42 (72.4)	17 (27.6)	0.13
Female	24 (57.1)	18 (42.9)	
*Daycare going children*			
Yes	10 (52.6)	9 (47.4)	0.18
No	56 (69.1)	25 (30.9)	
*Skin infections (within 6 months)*			
Yes	12 (75.0)	4 (25.0)	0.56
No	54 (64.3)	30 (35.7)	
*Hospital admission(within 6 months)*			
Yes	26 (61.9)	16 (38.1)	0.52
No	40 (69.0)	18 (31.0)	
*Hospital visits (within 6 months)*			
Yes	41 (60.3)	27 (39.7)	0.11
No	25 (78.1)	7 (21.9)	
*Volunteers in health Institution*			
Yes	3 (50.0)	3 (50.0)	0.40
No	63 (67.0)	31 (33.0)	
*Pork handle (within 6 months)*			
≥1times per week	40 (71.4)	16 (28.6)	0.21
Do not handle	26 (59.1)	18 (40.9)	
*Poultry handle (within 6 months)*			
≥1times per week	52 (71.2)	21 (28.8)	0.09
Do not handle	14 (51.8)	13 (48.2)	
*Length of stay in the U.S.*			
Less than 5 years	45 (70.3)	19 (29.7)	0.27
5 or more years	21 (58.3)	15 (41.6)	
*Family size*			
≤5 members	29 (64.4)	16 (35.6)	0.83
>5 members	37 (67.3)	18 (32.7)	
*Occupation*			
Daily wages labor	35 (74.5)	12 (25.5)	0.13
Others	31 (58.5)	22 (41.5)	
*Antibiotics use (within 6 months)*			
Yes	7 (58.3)	5 (41.7)	0.52
No	59 (67.1)	29 (32.9)	
*Skin infections (within 6 months)*			
Yes	12 (75.0)	4 (25.0)	0.56
No	54 (64.3)	30 (35.7)	
*Participated in Sports (within 6 months)*			
Yes	26 (62.0)	16 (38.0)	0.52
No	40 (69.0)	18 (31.0)	

**Table 3:** Exposure variables in association with *S. aureus* colonization in Nepal.

Variables (n=100)	SA+ (%)	SA – (%)	P-value
*Gender*			
Female	34 (47.9)	37 (52.1)	0.27
Male	10 (34.5)	19 (65.5)	
*Religion*			
Kirat	37 (46.9)	42 (53.1)	0.32
Others	7 (33.3)	14 (66.7)	
*Education*			
Formal Education	27 (45)	33 (55)	0.83
No formal Education	17 (42.5)	23 (57.5)	
*Marital status*			
Married	36 (41.9)	50 (58.1)	0.38
Others	8 (57.1)	6 (42.9)	
*Family size*			
≤5 members	26 (46.4)	30 (53.6)	0.68
>5 members	18 (41.0)	26 (59.0)	
*Daycare*			
Yes	11 (47.8)	12 (52.8)	0.81
No	33 (42.9)	44 (57.1)	
*Nearest farm*			
< 50meter	24 (41.4)	34 (58.6)	0.54
≥50meters	20 (47.6)	22 (52.4)	
*Hand wash frequency*			
After each work	39 (46.4)	45 (53.6)	0.28
Before meal	5 (31.2)	11 (68.8)	
Skin infections (within 6 months)			
Yes	16 (51.6)	15 (48.4)	0.38
No	28 (40.6)	41 (59.4)	
*Autoimmune disease (within 6 months)*			
Yes	9 (60.0)	6 (40.0)	0.17
No	34 (41.0)	49 (59.0)	
*Ear Infection(within 6 months)*			
Yes	13 (39.0)	20 (61.0)	0.51
No	31 (46.0)	36 (54.0)	
*Antibiotics use (within 6 months)*			
Yes	8 (32.0)	17 (68.0)	0.16
No	36 (48.0)	39 (52.0)	
*Skin disease (within 6 months)*			
Yes	16 (51.6)	15 (48.4)	0.30
No	28 (40.6)	41 (59.4)	
*Hospital admission(within 6 months)*			
Yes	3 (30.0)	7 (70.0)	0.34
No	41 (45.6)	49 (54.4)	
*Hospital visits (within 6 months)*			
Yes	4 (25.0)	12 (75.0)	0.09
No	40 (47.6)	44 (52.4)	
*Volunteers in health Institution*			
Yes	12 (42.9)	16 (57.1)	0.88
No	32 (44.4)	40 (55.6)	
*Pork handle (within 6 months)*			
Yes	7 (31.8)	15 (68.2)	0.19
No	37 (47.4)	41 (52.6)	
*Poultry handle (within 6 months)*			
Yes	24 (40.0)	36 (60.0)	0.32
No	20 (50.0)	20 (50.0)	

**Table 4:** Molecular characteristics of selected *S. aureus* isolates from NEO (N=38).

Isolates	Source	*mecA*	PVL	AST	*spa*	MLST
27NEOT	Throat	–	–	P	t002	ST5
89NEOT	Throat	–	+	P, G, C, L, T	t005	ST672
33NEON	Nose	+	–	P, O, E	t008	ST8
3NEOT	Throat	–	–	P, C, L, TS	t084	ST2885
11NEON	Throat	–	–	P	t085	ST845
38NEOT	Throat	–	–	P, TS	t091	ST789
9NEOT	Throat	–	–	P, T	t104	ST8
68NEOT	Throat	–	–	P	t10559	ST2733
23NEOT	Throat	–	+	P, TS	t1094	ST2112
80NEOT	Throat	–	+	P, C, L	t11906	ST2884
63NEOT	Throat	–	–	P, E, CL	t122	ST2102
8NEON	Nose	–	–	P	t127	ST573
69NEON	Nose	–	–	P	t131	ST1290
72NEOT	Throat	–	–	P	t164	ST20
48NEOT	Throat	–	–	P	t1654	ST667
30NEOT	Throat	–	–	P, T	t1818	ST96
1NEOT	Throat	–	+	P, C, L, TS	t1839	ST3206
82NEOT	Throat	–	–	P	t190	ST8
70NEOT	Throat	–	–	P, TS	t1931	ST1
84NEOT	Throat	–	–	P	t2119	ST2871
88NEOT	Throat	–	–	P	t2379	ST5
39NEOT	Throat	–	+	P, C, L, E, CL	t2663	ST2233
18NEOT	Throat	–	–	P	t273	ST1
1NEON	Nose	–	+	P, C, L, TS	t345	ST3206
59NEOT	Throat	–	–	P	t4371	ST5
4NEOT	Throat	–	–	P, TS	t442	ST5
13NEOT	Throat	–	–	P, C, L, TS	t491	ST199
9NEON	Nose	–	–	P, C, L, TS	t521	ST96
96NEOT	Throat	–	–	P	t6127	ST8
50NEOT	Throat	–	–	P	t616	ST944
12NEOT	Throat	–	–	P	t688	ST5
8NEOT	Throat	–	–	P	t7264	ST5
55NEOT	Throat	–	–	P	t774	ST15
79NEOT	Throat	–	–	P	t803	ST15
28NEOT	Throat	–	–	P	t818	ST5
29NEOT	Throat	–	–	P	t878	ST2849
58NEOT	Throat	–	–	P, G, E, CL	t934	ST80
16NEOT	Throat	–	–	P, C, L	t9432	ST2128

P, benzylpenicillin; C, ciprofloxacin; E, erythromycin; CL, clindamycin; G, gentamicin; T, tetracycline; TS, trimethoprim-sulfamethoxazole; L, levofloxacin; O, oxacillin; AST, antibiotic susceptibility testing. Inclusion of antibiotic name denotes resistance.

**Table 5:** Molecular characteristics of selected *S. aureus* isolates from Nepal (N=29).

Isolates	Source	*mecA*	PVL	AST	*Spa*	ST
68T5	Throat	–	–	P, TS	t002	ST5
26T5	Throat	–	–	P, C	t11917	ST72
71T5	Throat	–	–	P	t15579	ST2885
74N	Nose	–	–	P, C, L, E, CL	t15581	ST3206
19N	Nose	–	–	P	t008	ST8
101N	Nose	–	+	P, L, M, TS	t021	ST1482
102T	Throat	–	–	P, TS	t084	ST15
76T	Throat	–	–	P	t085	ST15
12T	Throat	–	–	P	t091	ST2081
24N	Nose	–	–	P, C, L	t1149	ST291
105T	Throat	–	+	P, G, C, L, E	t12219	ST672
83T	Throat	+	–	P, O, C, E, CL, Mi, T, R	t127	ST1
48T	Throat	–	–	P, G, E, CL	t1427	ST361
70T	Throat	–	–	P	t15578	ST2990
1T	Throat	–	–	P, E, CL, TS	t159	ST800
85T	Throat	–	–	P, G, TS	t164	ST20
67T	Throat	–	+	P, TS	t1839	ST573
65T	Throat	–	–	P, TS	t2119	ST2871
106T	Throat	–	+	P, G, C, L, E	t2663	ST2233
15T	Throat	+	–	P, O	t304	ST6
32N	Nose	–	+	P, TS	t311	ST5
42T	Throat	–	–	P, G, T	t3175	ST672
77N	Nose	+	+	P, O, CL, R	t345	ST3206
97T	Throat	–	–	P, E, CL	t376	ST80
82T	Throat	–	–	P, C	t4188	ST199
33T2	Throat	–	–	P, TS	t442	ST5
107T	Throat	–	–	P	t701	ST6
56T	Throat	–	–	P	t878	ST2849
109N	Nose	–	–	P, G, E, CL	t934	ST80

## Data Availability

Data are available upon request to the authors.

## Supplementary Materials

Supplementary Materials The materials provided were added to the manuscript to provide additional detail of molecular analyses of spa and multilocus sequence types found in samples from Northeast Ohio and Nepal.
